# Supplementary material for: USP49 potently stabilizes APOBEC3G protein by removing ubiquitin and inhibits HIV-1 replication
Source: eLife. 2019 Aug 9;8:e48318. doi: 10.7554/eLife.48318 (PMC6701944; doi:10.7554/eLife.48318)
Supplement: Supplementary file 1. [file elife-48318-supp1.docx]

**Supplementary File 1. The sequences of primers and probes**

| **Primer** | **Sequence (5’-3’)** |
| --- | --- |
| shUSP49-F | AATTGCCGTAATCATCGAGAGAAGACTCG  AGTCTTCTCTCGATGATTACGGCTTTTTTTAT |
| shUSP49-R | AAAAAAAGCCGTAATCATCGAGAGAAGACT  CGAGTCTTCTCTCGATGATTACGGC |
| Qpcr-Actin-f | CATGTACGTTGCTATCCAGGC |
| Qpcr-Actin-r | CTCCTTAATGTCACGCACGAT |
| Qpcr-GAPDH-F | GGAGCGAGATCCCTCCAAAAT |
| Qpcr-GAPDH-R | GGCTGTTGTCATACTTCTCATGG |
| Qpcr-USP18-F | CCTGAGGCAAATCTGTCAGTC |
| Qpcr-USP18-R | CGAACACCTGAATCAAGGAGTTA |
| Qpcr-USP41-F | GTATGTCACTGTGGGCCTCAT |
| Qpcr-USP41-R | ACAGGGGCACGTTGTACTTC |
| Qpcr-USP49-F | TGGTCTGGCCGTAATCATCG |
| Qpcr-USP49-R | CCTGCAGCAGTAAGGTTCCA |
| ddpcr-Psi-f | CAGGACTCGGCTTGCTGAAG |
| ddpcr-Psi-r | GCACCCATCTCTCTCCTTCTAGC |
| ddpcr-Psi probe | FAM-TTTTGGCGTACTCACCAGT-MGB |
| ddpcr-Env-f | AGTGGTGCAGAGAGAAAAAAGAGC |
| ddpcr-Env-r | GTCTGGCCTGTACCGTCAGC |
| ddpcr-Env-intact probe | VIC-CCTTGGGTTCTTGGGA-MGB |
| ddpcr-Env-hypermut probe | Unlabeled-CCTTAGGTTCTTAGGAGC-MGB |
